# Factors affecting the self-rated health of elderly individuals living alone: a cross-sectional study

**DOI:** 10.1186/s13104-017-2836-x

**Published:** 2017-10-26

**Authors:** Koji Yoshimitsu, Takayuki Tabira, Masatomo Kubota, Yuriko Ikeda, Kazuhiro Inoue, Yasuaki Akasaki

**Affiliations:** Department of Basic Occupational Therapy, Faculty of Medicine, School of Sciences, Kagoshima University, 8-35-1 Sakuragaoka, Kagoshima, 890-8544 Japan

**Keywords:** Self-rated health, Elderly individuals living alone, Activities of daily living, Instrumental activities of daily living

## Abstract

**Objective:**

In Japan, the number of elderly individuals living alone is continuing to increase as society ages. Although studies have considered quality of life, life expectancy, and gender differences in elderly individuals living alone, only a few have considered the health and lifestyle of these individuals. Therefore, we aimed to investigate the factors that affect the self-rated health of elderly individuals living alone to understand this group and how best to offer them support.

**Results:**

We include 113 individuals. There was a significant difference (P < 0.05) in some characteristics (e.g., age, chronic illness, frequency of hospital visits in 1 month, and caregivers), activities of daily living (e.g., motor tasks), and instrumental activities of daily living (e.g., household affairs, heavy housework, outdoor household, and outdoor activities).

## Background

The Japanese elderly population (age ≥ 65 years) has reached a record high of 33 million. This constitutes the highest number of elderly men and women as a proportion of the population when compared with other developed nations since 2005 [1]. In tandem with this, the average number of individuals per general household is expected to decrease from 2.42 in 2010 to 2.20 in 2035, with one-in-four households expected to be home to an elderly individual who lives alone [2]. Also, the number of individuals living with their offspring has been decreasing each year, and by 2015 there was an estimated 7 million households with individuals aged ≥ 65 years who were living alone [3].

The increase in the number of elderly individuals is a recognized social concern, and in recent years, there have been several studies regarding elderly individuals living alone. However, most of these examined quality of life [4–6], the prognosis of illness or impairment [7], or gender differences [8, 9]. Although aging cannot be prevented, better understanding by local residents can help them feel healthy despite functional deterioration and some diseases. To date, few studies have investigated the health and lifestyle of elderly individuals living alone. Although many elderly individuals who live alone do so in the general community, their characteristics and lifestyles remain poorly understood. Self-rated health (SRH), defined as perceived health in comparison to another age-matched individual [10], is an important consideration in this group.

In this study, we aimed to clarify the factors associated with SRH among elderly individuals living alone, and which factors positively contribute to the basic understanding, so that we can support these individuals to have a better SRH. Therefore, we clarified the characteristics, activities of daily living (ADLs), and instrumental ADL (IADLs) of elderly individuals living alone, and how these relate to SRH.

## Methods

### Study design

We conducted a cross-sectional study by face-to-face interviews. Study participants were divided into two groups based on SRH (i.e., a good SRH group and a poor SRH group), and statistically significant differences in characteristics, ADLs, and IADLs were examined.

### Study setting and participants

Using systematic sampling, we randomly selected elderly individuals living alone within a rural area of Kagoshima prefecture (Town A). This region has an elderly population rate of 35.1%, which is above the national average. Individuals were included if they were aged ≥ 65 years, had lived alone for at least 1 year, and had no communication impairments. Individuals who agreed to participate in the study were visited at home and interviewed by the authors in the presence of a member of staff from the Senior Citizen’s Welfare Division of the local town hall.

### Participant characteristics

The interviews were performed using a structured questionnaire. The questions included the following: length of time spent living alone, marital status, certification of need for long-term care or support, the presence of chronic illness, frequency of hospital visits per month, the presence of emotional support, the presence of functional support, supporter characteristics, and SRH. Participants graded their SRH on a 4-point scale as good, quite good, fair, or poor. Those who responded “good” or “quite good” were included in the good SRH group, and those who responded “fair” or “poor” were included in the poor SRH group.

### ADL analysis

Activities of daily living were evaluated using the functional independence measure (FIM). This measure consisted of 18 items, including 13 motor tasks and 5 cognitive tasks, with each task evaluated regarding the participant’s level of independence on a 7-point scale from 1 (total assistance) to 7 (complete independence).

### IADL evaluation

Instrumental activities of daily livings were evaluated using the Frenchay activities index (FAI). The FAI is a measure of IADLs that was developed by Holbrook and Skilbeck [11] to evaluate stroke patients [12]. However, Matsunaga et al. developed a Japanese version [13, 14] that is used to evaluate IADLs in the management of strokes and subacute myelo-optic neuropathy [8, 15, 16], as well as in elderly individuals in general [13, 17]. The FAI consists of 15 items, including five items for household affairs, three items for outdoor household activities, four items for outdoor activities, two items for hobbies, and one item for work. The frequency of the participant’s activity over the past 3 or 6 months is then evaluated according to a 4-point scale from 0 (inactive) to 3 (highly active).

### Statistical analysis

Various data obtained from the two groups were analyzed using IBM SPSS for Windows. Basic analyses were by a *t* test, with age, length of time living alone, and the frequency of medical examination per month expressed as mean ± standard deviation (median, minimum–maximum). The number of participants was expressed as a percentage and analyzed by Chi square tests. Finally, the ADLs and IADLs were expressed as mean ± standard deviation (median, minimum–maximum) and analyzed by the Mann–Whitney *U* test. Differences with a P value < 0.05 were considered statistically significant.

## Results

### Characteristics of elderly participants

We included 113 elderly participants in the survey, and their characteristics are summarized in Table 1. As shown, most participants were elderly and most were widows. The average duration of living alone was more than 10 years, with about half being certified as receiving long-term care insurance, and most being hospitalized for the treatment of chronic diseases. Most also received emotional support from children, and more than half received functional support. Fairly good was the most frequent SRH rating, followed by fairly poor.

**Table 1:** Characteristics of subject and relationships between good SRH and poor SRH

	Overall (n = 113)	Good SRH (n = 77)	Poor SRH (n = 36)	P value
Age (years)				0.008 (**)^a^
Mean ± SD (median, range)	83.3 ± 5.5 (84, 65–96)	84.3 ± 5.0 (85, 73–96)	81.4 ± 6.1 (82, 65–92)	
Gender, n (%)				1.00^b^
Female	97 (85.8%)	66 (85.7%)	31 (86.1%)	
Male	16 (14.2%)	11 (14.3%)	5 (13.9%)	
Length of time lived alone (years)				0.48^a^
Mean ± SD (median, range)	12.9 ± 11.3 (10, 1–54)	12.4 ± 9.6 (10, 1–54)	14.2 ± 14.4 (10, 1–50)	
Marital status, n (%)				0.12^b^
Widow (er)	96 (85%)	69 (89.6%)	27 (75.0%)	
Divorce	10 (8.8%)	5 (6.5%)	5 (13.9%)	
Single	7 (6.2%)	3 (3.9%)	4 (11.1%)	
Certification of needed long-term care or support, n (%)				0.10^b^
Not certified	58 (51.3%)	44 (57.1%)	14 (38.9%)	
Support level 1	17 (15.0%)	13 (16.9%)	4 (5.4%)	
Support level 2	13 (11.5%)	6 (7.8%)	7 (19.4%)	
Care level 1	8 (7.1%)	6 (7.8%)	2 (5.6%)	
Care level 2	12 (10.6%)	5 (6.5%)	7 (19.4%)	
Care level 3	5 (4.4%)	3 (3.9%)	2 (5.6%)	
Care level 4	0 (0%)	0 (0%)	0 (0%)	
Care level 5	0 (0%)	0 (0%)	0 (0%)	
Chronic malady, n (%)				0.03 (*)^a^
Presence	104 (92.0%)	68 (88.3%)	36 (100.0%)	
Absence	9 (8.0%)	9 (11.7%)	0 (0.0%)	
Frequency of hospital visit per 1 month				0.000 (***)^a^
Mean ± SD (median, range)	4.1 ± 4.0 (2, 0–20)	3.0 ± 2.8 (2, 0–12)	6.5 ± 4.9 (5.5, 1–20)	
Emotional support, n (%)				0.000 (***)^b^
Presence	104 (92.0%)	76 (98.7%)	28 (77.8%)	
Absence	9 (8%)	1 (1.3%)	8 (22.2%)	
Instrumental support, n (%)				0.70^b^
Presence	75 (66.4%)	52 (67.5%)	23 (63.9%)	
Absence	38 (33.6%)	25 (32.5%)	13 (36.1%)	
Caregivers, n (%)				0.04 (*)^b^
Children	81 (77.9%)	63 (84.0%)	18 (62.1%)	
Blood relatives	14 (13.5%)	8 (10.7%)	6 (20.7%)	
Neighbors	9 (8.7%)	4 (5.3%)	5 (17.2%)	
Self-rated health, n (%)				
Good	5 (4.4%)	5 (6.5%)		
Fairly good	72 (63.7%)	72 (93.5%)		
Fairly poor	32 (28.3%)		32 (88.9%)	
Poor	4 (3.5%)		4 (11.1%)	

Ages, length of time lived alone, frequency of hospital visit per 1 month presented as mean, standard deviation (median, min–max), and differences were examined using *t*-test. The others presented as number (percentages), and differences were examined using Chi square test

* P < 0.05; ** P < 0.01; *** P < 0.001

^a^ *t*-test

^b^Chi square test

Participants were divided into two groups according to the degree of SRH: a good SRH group (n = 77) and a poor SRH group (n = 36). The good SRH group tended to be older (P < 0.01), have a lower rate of chronic illnesses (P < 0.05), have a lower frequency of hospital visits per month (P < 0.001), receive a higher rate of emotional support (P < 0.001), and have a difference in the ratio of supporters (P < 0.05) compared with the poor SRH group.

### ADL of elderly individuals living alone

The ADL results are summarized in Table 2. The mean total FIM score in the good SRH group was 116.1 ± 10.3 (120, 83–126) points, with 84.0 ± 7.8 (87, 61–91) points for motor tasks, and 32.1 ± 3.6 (34, 22–35) points for cognitive tasks.

**Table 2:** Score of the FIM and relationships between good SRH and poor SRH

	Overall (n = 113)	Good SRH (n = 77)	Poor SRH (n = 36)	P value
Motor tasks (7–91)	83.1 ± 8.1 (86, 60–91)	84.0 ± 7.8 (87, 61–91)	81.2 ± 8.3 (83.5, 60–91)	0.03 (*)
Eating (1–7)	6.8 ± 0.5 (7, 5–7)	6.8 ± 0.5 (7, 5–7)	6.8 ± 0.5 (7, 5–7)	1.00
Grooming (1–7)	6.7 ± 0.7 (7, 2–7)	6.8 ± 0.4 (7, 6–7)	6.5 ± 1.0 (7, 2–7)	0.1
Bathing (1–7)	6.3 ± 1.1 (7, 2–7)	6.4 ± 1.0 (7, 2–7)	6.0 ± 1.3 (6, 3–7)	0.06
Upper body dressing (1–7)	6.6 ± 0.7 (7, 4–7)	6.6 ± 0.6 (7, 5–7)	6.4 ± 0.8 (7, 4–7)	0.27
Under body dressing (1–7)	6.6 ± 0.6 (7, 4–7)	6.6 ± 0.5 (7, 5–7)	6.4 ± 0.7 (7, 4–7)	0.16
Toileting (1–7)	6.5 ± 0.6 (7, 4–7)	6.6 ± 0.5 (7, 5–7)	6.3 ± 0.7 (6, 4–7)	0.04 (*)
Bladder management (1–7)	6.6 ± 0.7 (7, 3–7)	6.6 ± 0.7 (7, 3–7)	6.6 ± 0.6 (7, 3–7)	0.67
Bowel management (1–7)	6.7 ± 0.5 (7, 4–7)	6.7 ± 0.6 (7, 3–7)	6.7 ± 0.5 (7, 5–7)	0.73
Bed to chair transfer (1–7)	6.3 ± 0.8 (6, 3–7)	6.4 ± 0.8 (7, 3–7)	6.2 ± 0.7 (6, 3–7)	0.08
Toilet transfer (1–7)	6.4 ± 0.7 (6, 3–7)	6.4 ± 0.7 (7, 3–7)	6.3 ± 0.5 (6, 6–7)	0.11
Shower transfer (1–7)	6.0 ± 1.3 (6, 1–7)	6.1 ± 1.2 (6, 1–7)	5.7 ± 1.5 (6, 1–7)	0.07
Locomotion (1–7)	6.1 ± 1.0 (6, 2–7)	6.2 ± 0.9 (6, 3–7)	5.9 ± 1.2 (6, 2–7)	0.22
Stairs (1–7)	5.6 ± 1.3 (6, 1–7)	5.7 ± 1.3 (6, 1–7)	5.3 ± 1.5 (6, 1–7)	0.16
Cognitive tasks (5–35)	31.8 ± 4.4 (34, 10–35)	32.1 ± 3.6 (34, 22–35)	31.2 ± 5.7 (33, 10–35)	0.61
Cognitive comprehension (1–7)	6.4 ± 1.0 (7, 2–7)	6.4 ± 0.9 (7, 3–7)	6.3 ± 1.2 (7, 2–7)	0.66
Expression (1–7)	6.5 ± 0.9 (7, 2–7)	6.5 ± 0.7 (7, 4–7)	6.3 ± 1.2 (7, 2–7)	0.54
Social intercourse (1–7)	6.4 ± 0.9 (7, 2–7)	6.5 ± 0.8 (7, 4–7)	6.3 ± 1.2 (7, 2–7)	0.73
Problem solving (1–7)	6.3 ± 1.0 (7, 2–7)	6.4 ± 0.9 (7, 3–7)	6.1 ± 1.2 (6, 2–7)	0.13
Memory (1–7)	6.3 ± 1.0 (7, 2–7)	6.3 ± 1.0 (7, 3–7)	6.3 ± 1.2 (7, 2–7)	0.91
Total scores (18–126)	114.9 ± 10.9 (118, 78–126)	116.1 ± 10.3 (120, 83–126)	112.4 ± 11.9 (115.5, 78–126)	0.07

FIM scores presented as mean, standard deviation (median, minimal–maximum), and differences were examined using Mann–Whitney U test

* P < 0.05

The mean total FIM score in the poor SRH group was 112.4 ± 11.9 (115.5, 78–126) points, with 81.2 ± 8.3 (83.5, 60–91) points for motor tasks, and 31.2 ± 5.7 (33, 10–35) points for cognitive tasks. In addition, the good SRH group had higher scores (indicating independence) for motor tasks (P < 0.05) compared with the poor SRH group, and scores were particularly high for toileting (P < 0.05).

### IADLs of elderly individuals living alone

The IADL data are shown in Table 3. The mean total FAI score in the good SRH group was 23.6 ± 9.2 (24, 3–40) points, with 11.5 ± 3.8 (13, 0–15) points for household affairs, 4.1 ± 2.5 (4, 0–9) points for outdoor household activities, 1.2 ± 1.2 (1, 0–8) points for outdoor activities, 2.3 ± 2.0 (3, 0–6) points for hobbies, and 0.1 ± 0.5 (0, 0–3) points for work.

**Table 3:** Score of the FAI Japanese version and relationships between good SRH and poor SRH

	Overall (n = 113)	Good SRH (n = 77)	Poor SRH (n = 36)	P value
Household affairs (0–15)	10.8 ± 4.1 (12, 0–15)	11.5 ± 3.8 (13, 0–15)	9.2 ± 4.5 (10, 0–15)	0.002 (**)
Preparing meals (0–3)	2.5 ± 1.0 (3, 0–3)	2.6 ± 0.9 (3, 0–3)	2.4 ± 1.2 (3, 0–3)	0.47
Washing up (0–3)	2.7 ± 0.8 (3, 0–3)	2.8 ± 0.7 (3, 0–3)	2.4 ± 1.1 (3, 0–3)	0.15
Washing clothes (0–3)	2.6 ± 0.9 (3, 0–3)	2.7 ± 0.8 (3, 0–3)	2.4 ± 1.2 (3, 0–3)	0.31
Light housework (0–3)	2.0 ± 1.2 (2, 0–3)	2.2 ± 1.1 (3, 0–3)	1.5 ± 1.2 (1, 0–3)	0.004 (**)
Heavy housework (0–3)	1.0 ± 1.2 (1, 0–3)	1.3 ± 1.2 (1, 0–3)	0.5 ± 1.0 (0, 0–3)	0.000 (***)
Outdoor household (0–9)	3.5 ± 2.7 (3, 0–9)	4.1 ± 2.5 (4, 0–9)	2.3 ± 2.6 (1, 0–8)	0.000 (***)
Local shopping (0–3)	1.8 ± 1.4 (3, 0–3)	2.0 ± 1.3 (3, 0–3)	1.2 ± 1.4 (0, 0–3)	0.005 (**)
Gardening (0–3)	1.3 ± 1.2 (1,0–3)	1.5 ± 1.1 (2,0–3)	0.8 ± 1.0 (0,0–3)	0.000 (***)
Household/car maintenance (0–3)	0.4 ± 0.8 (0, 0–3)	0.5 ± 0.8 (0, 0–3)	0.3 ± 0.7 (0, 0–3)	0.09
Outdoor activities (0–12)	1.3 ± 1.3 (1, 0–8)	1.2 ± 1.2 (1, 0–8)	1.5 ± 1.6 (1, 0–8)	0.12
Social occasions (0–3)	1.4 ± 1.3 (1, 0–3)	1.5 ± 1.4 (1, 0–3)	1.3 ± 1.3 (1, 0–3)	0.52
Walking outside (0–3)	1.5 ± 1.3 (1, 0–3)	1.4 ± 1.4 (1, 0–3)	1.5 ± 1.3 (1, 0–3)	0.92
Driving car/bus travel (0–3)	2.4 ± 1.0 (3, 0–3)	2.6 ± 0.9 (3, 0–3)	2.1 ± 1.3 (3, 0–3)	0.04 (*)
Travel outings/car rides (0–3)	0.1 ± 0.4 (0, 0–3)	0.1 ± 0.4 (0, 0–3)	0.1 ± 0.3 (0, 0–1)	0.72
Hobby (0–6)	2.1 ± 2.0 (2, 0–6)	2.3 ± 2.0 (3, 0–6)	1.7 ± 2.0 (1, 0–6)	0.09
Actively pursuing hobby (0–3)	1.2 ± 1.4 (0, 0–3)	1.4 ± 1.4 (1, 0–3)	1.0 ± 1.4 (0, 0–3)	0.16
Reading books (0–3)	0.9 ± 1.2 (0, 0–3)	0.9 ± 1.2 (0, 0–3)	0.7 ± 1.2 (0, 0–3)	0.33
Work (0–3)				
Gainful work (0–3)	0.1 ± 0.5 (0, 0–3)	0.1 ± 0.5 (0, 0–3)	0.1 ± 0.5 (0, 0–3)	0.41
Total score (0–45)	21.9 ± 9.7 (21, 2–42)	23.6 ± 9.2 (24, 3–40)	18.3 ± 9.9 (18.5, 2–42)	0.006 (**)

FAI scores presented as mean, standard deviation (median, min–max), and differences were examined using Mann–Whitney U test

* P < 0.05; ** P < 0.01; *** P < 0.001

The mean total FAI score in the poor SRH group was 18.3 ± 9.9 (18.5, 2–42) points, with 9.2 ± 4.5 (10, 0–15) points for household affairs, 2.3 ± 2.6 (1, 0–8) points for outdoor household activities, 1.5 ± 1.6 (1, 0–8) points for outdoor activities, 1.7 ± 2.0 (1, 0–6) points for hobbies, and 0.1 ± 0.5 (0, 0–3) points for work.

The good SRH group had higher total scores (P < 0.01) than the poor SRH group in moderate items, with high scores for household affairs (P < 0.01) and outdoor household activities (P < 0.01). For minor items, high scores were also obtained for light housework (P < 0.01), heavy housework (P < 0.01), local shopping (P < 0.01), and driving a car or traveling by bus (P < 0.05).

## Discussion

### Characteristics

There are contradictory reports concerning age. For example, one study rejected the relationship between SRH and mortality, which are similar variables in elderly populations [18]. Other studies have reported poor SRH as a strong predictor of mortality [19], or that people with a poor SRH had a twofold higher risk of mortality than people with good SRH [20]. The number of elderly individuals with health or ADL problems increases with age, and among elderly individuals, loneliness has been shown to be a predictor of functional decline [21]. However, other research [22] indicates that older people tend to adapt to various age-related conditions. In the present study, the fact that the eldest group exhibited good SRH supports this view. It seems that extremely elderly individuals living alone may be able to adapt to independent living because they have a good SRH.

Japan’s long-term care insurance services are offered when people age 65 years or older need care and support or people aged 40–64 years develop age-related diseases that require care and support. In this system, insured persons are assessed for mental and physical condition, reviewed by a doctor, and certified as “self-supporting”, support level 1–2, or care level 1–5. In a study of elderly individuals [23], it was reported that the presence or absence of certification for requiring long-term care or support correlated with the SRH score; however, we could not confirm these results by the level of care needed. Our results (i.e., the association with the presence or absence of chronic illnesses and the frequency of hospital visits per month) are consistent with studies showing correlations for SRH with medical history, chronic illness, and number of chronic illnesses [19, 24, 25].

Regarding the presence or absence of emotional support in Japanese participants, one study reported that the SRH of the parent was clearly correlated with support received from their offspring [26], similar to the findings of our study. However, a study conducted in Spain demonstrated that functional, rather than emotional, support led to good SRH in widowers (husband) [27], which conflicts with our results. Studies on individuals living alone have reported different results depending on the socioeconomic status and culture, which may account for the lack of consistency. Based on these results, important facilitators of elderly individuals having good SRH when living alone are as follows: (1) they either have no chronic illness or a chronic illness that does not require frequent hospital visits; (2) they have access to emotional support; and (3) the primary supporter/caregiver is his or her offspring.

### The role of ADLs

Research has shown that independence of ADLs is facilitated by having good physical abilities, which is also an important variable of better perceived health, indirectly supporting the results of this study [28]. For most elderly individuals living alone, basic ADLs were not problematic or associated with SRH, but the fact that independent toileting was the most important factor for most is consistent with the results of an earlier study [29]. The same study noted that motor tasks of inpatients were able to return to living alone were significantly improved. Together with our results, these findings suggest that physical ability and independent toileting are important for personal dignity and promote good SRH in elderly individuals living alone.

### The role of IADLs

It has become apparent that, in general, the elderly living alone do not intensively pursue outdoor activities or hobbies but instead seem to realize their health by performing housework. They view housekeeping, which requires physical effort, to be particularly important. In the gender-related difference in Japanese lifestyle, females are exclusively involved with housework [13]. There is a possibility that the traditional lifestyle pattern peculiar to Japan influenced this study which female occupy most.

Important factors for good SRH in elderly individuals living alone include the following: (1) they do not have chronic illnesses, or if present, they do not require frequent hospital visits; (2) they can obtain emotional support from offspring; and (3) they can maintain motor function in daily life; (4) they can perform toileting independently; and (5) they can perform most habitual activities independently (e.g., housework and driving a car or traveling by bus).

## Conclusions

It was notable that good SRH was associated with significantly more advanced age compared with poor SRH. Good SRH was also associated with less certification of the need for long-term care, fewer chronic illnesses, fewer hospital visits, more emotional support, and more support from offspring. Significantly higher independence was also noted for FIM motor tasks in those with good SRH compared with poor SRH, particularly for toileting, and there was a significant difference in the total FAI scores between the two groups. It was also noted that engaging in mild- and moderate-intensity activities was significantly different between the groups. Therefore, we conclude that understanding the lifestyles of elderly individuals who live alone and the factors that affect their SRH are important when offering support.

### Limitations

A limitation of this study is that the participants were selected from only one rural area of Japan. The 113 participants accounted for only 12.5% of all elderly individuals living alone in Town A, so we cannot conclude that it is representative of all elderly individuals living alone. A verification power analysis was performed, as instructed which indicated powers (1 − β) of 0.689, 0.623, and 0.889 for t tests, Chi square tests, and Mann–Whitney U tests, respectively. It was possible that some items could not be detected in the attributes section due to the small sample size. Although no related articles were identified, we concluded that the ADLs or IADLs of elderly individuals living alone are likely to be affected by aging and that the fluctuation of SRH may be influenced by depression, which must be taken into consideration. Furthermore, the results were probably affected by the unique culture of Japan.

## Abbreviations

SRH: self-rated health
ADL: activities of daily living
IADL: instrumental activities of daily living
FIM: functional independence measure
FAI: Frenchay activities index
